# Health Status Assessment of Passenger Ropeway Bearings Based on Multi-Parameter Acoustic Emission Analysis

**DOI:** 10.3390/s25144403

**Published:** 2025-07-15

**Authors:** Junjiao Zhang, Yongna Shen, Zhanwen Wu, Gongtian Shen, Yilin Yuan, Bin Hu

**Affiliations:** Key Laboratory of Nondestructive Testing and Evaluation, China Special Equipment Inspection & Research Institute, State Administration for Market Regulation, Beijing 100029, China; zhangjunjiao@csei.org.cn (J.Z.); shenyongna@csei.org.cn (Y.S.); wuzhanwen@csei.org.cn (Z.W.); hubin@csei.org.cn (B.H.)

**Keywords:** acoustic emission, rolling bearing, condition monitoring, signal characteristic, passenger ropeway

## Abstract

**Highlights:**

- The health status of passenger ropeway bearings is studied using multi-parameter AE analysis.
- Resonant AE sensors outperform broadband sensors in defect detection.
- The laboratory research results have been successfully applied to field testing of passenger ropeway rolling bearings for two years.
- A novel LLM-based approach achieves automated bearing wear detection.

**What are the main findings?**

- Defective bearings exhibit periodic RMS peaks and elevated mean values.
- Field tests confirm AE’s effectiveness in detecting early-stage bearing damage.
- The pre-trained Paligemma LLM model demonstrates superior accuracy in wear feature identification.

**What is the implication of the main finding?**

- Offers a practical solution for preventive maintenance in passenger ropeway systems.
- Demonstrates the successful transition from laboratory research to practical field applications.

**Abstract:**

This study presents a comprehensive investigation of acoustic emission (AE) characteristics for condition monitoring of rolling bearings in passenger ropeway systems. Through controlled laboratory experiments and field validation across multiple operational ropeways, we establish an optimized AE-based diagnostic framework. Key findings demonstrate that resonant VS150-RIC sensors outperform broadband sensors in defect detection, showing greater energy response at characteristic frequencies for inner race defects. The RMS parameter emerges as a robust diagnostic indicator, with defective bearings exhibiting periodic peaks and higher mean RMS values. Field tests reveal progressive RMS escalation preceding visible damage, enabling predictive maintenance. Furthermore, we develop a novel Paligemma LLM model for automated wear detection using AE time-domain images. The research validates the AE technology’s superiority over conventional vibration methods for low-speed bearing monitoring, providing a scientifically grounded approach for safety-critical ropeway maintenance.

## 1. Introduction

Passenger ropeways, including aerial tramways, gondolas, and chairlifts, play a crucial role in China’s transportation infrastructure, particularly in mountainous regions and popular tourist destinations. With the rapid expansion of China’s tourism industry and the increasing demand for eco-friendly transport solutions, the number of passenger ropeways has grown substantially in recent years. According to the survey, by the end of 2024, there were over 1160 passenger ropeways in operation nationwide, serving millions of passengers annually. However, due to their exposure to extreme environmental conditions—such as heavy loads, temperature variations, and continuous operation—ensuring the structural integrity and mechanical reliability of these systems remains a critical challenge [1]. Any failure in key components, particularly rolling bearings, can lead to severe accidents, making proactive maintenance and condition monitoring essential for operational safety.

Rolling bearings are among the most critical mechanical components in passenger ropeway systems, supporting rotating shafts and reducing friction between moving parts. A typical rolling bearing consists of four primary elements: the inner race, outer race, rolling elements (balls or rollers), and the cage (retainer). The inner and outer races provide smooth rolling surfaces, while the rolling elements distribute loads evenly to minimize wear. The cage maintains proper spacing between the rolling elements to prevent contact and excessive friction. Due to the high mechanical stresses and cyclic loading experienced during operation, rolling bearings are susceptible to various failure modes, including fatigue spalling, pitting, and cage fracture [2]. Given that bearing failures can lead to catastrophic breakdowns in ropeway systems, developing effective diagnostic techniques for early fault detection is crucial for preventing unplanned downtime and ensuring passenger safety.

Several traditional techniques have been widely employed for rolling bearing fault detection, each with its advantages and limitations. Vibration analysis remains the most commonly used method, where accelerometers measure vibration signals to identify characteristic fault frequencies in time, frequency, and time–frequency domains [3]. Another approach is temperature monitoring, which tracks bearing temperature fluctuations to detect abnormal friction or lubrication failure [4]. Additionally, oil debris analysis examines wear particles in lubricating oil to assess bearing degradation trends [5]. While these methods have proven effective in many industrial applications, they often require direct sensor installation and may not detect early-stage defects before significant damage occurs. Furthermore, in ropeway systems, where bearings operate at relatively low speeds, conventional vibration-based techniques may lack sufficient sensitivity [6,7], necessitating alternative monitoring approaches.

Acoustic emission (AE) is a non-destructive testing technique that captures high-frequency stress waves (typically 20 kHz–1 MHz) generated by material deformation, crack propagation, or friction [8]. Unlike vibration analysis, which measures structural responses to faults, AE directly detects the energy released from active defects, making it highly sensitive to incipient damage. This method is particularly advantageous for monitoring slow-speed and heavily loaded bearings, where traditional vibration signals may be weak. Additionally, AE signals serve as carriers of fault information. Through analysis and processing of the acquired rolling bearing AE signals, various types of faults can be identified [9,10]. Passenger ropeway bearings may develop faults during operation due to various reasons such as improper assembly, inadequate lubrication, corrosion, and overloading. Even with correct installation, proper lubrication, and normal maintenance, prolonged operation can still lead to fatigue spalling and wear that eventually render the bearings inoperable. Operating under prolonged low-speed and heavy-load conditions, passenger ropeway bearings commonly exhibit failure modes characterized by fatigue spalling and wear. The ability to detect early-stage faults before they evolve into critical failures makes AE a promising tool for predictive maintenance in passenger ropeway systems.

Schnabel et al. [11] investigated the application of AE technology in detecting plastic deformation caused by particle contamination in rolling element bearings. The study revealed that at low speeds (≤100 rpm), AE signals dominated by plastic deformation induced by particle contamination exhibited significantly higher RMS values. In contrast, at high speeds (≥300 rpm), the amplitude of elastic waves increased quadratically with rotational speed, completely masking the plastic deformation signals. Hase [12] conducted experiments on thrust ball bearings by artificially creating defects on the raceway surfaces to simulate the fatigue crack initiation process, employing AE technology for monitoring. He classified the bearing damage progression into three distinct phases, with high-frequency AE signals progressively increasing as fatigue cracks developed. During the final crack propagation stage, the AE signal amplitude exhibited a sharp increase (>1.5 V) with a prominent high-frequency peak at 0.3 MHz becoming clearly detectable.

Aasi et al. [13] fabricated defects of 0.1 mm and 0.6 mm on the inner and outer races of angular contact bearings, collected AE signals under varying loads and rotational speeds using AE technology, and extracted 18 time-domain features from the AE signals to compare the sensitivity of these features under different defect sizes, loads, and speeds. His key innovation lies in proposing a rapid diagnostic framework based on time-domain features, enabling the distinction of defect types and sizes without the need for complex frequency-domain analysis. Chen et al. [14] introduced a multimodal signal fusion method that combines vibration and AE signals to enhance the diagnostic accuracy of bearing faults. Similar studies abound, with extensive research employing various deep learning methods for the classification, intelligent recognition, or prediction of rolling bearing damage. However, most of these studies [15,16,17,18] remain confined to laboratory settings. While highly valuable, their findings have yet to be adapted for practical field applications.

This research achieved AE monitoring of passenger ropeway rolling bearings through comprehensive laboratory experiments and field testing. A specialized test rig simulating actual ropeway operating conditions was established in the laboratory, operating at low rotational speeds. Sequential AE tests were conducted on both defect-free rolling bearings and those with inner race defects. Parameter analysis and waveform spectrum analysis were employed to contrast the AE characteristics between normal and defective bearings. The AE features acquired from laboratory studies were then applied in field tests on two operational ropeways. Furthermore, based on extensive field data, a pre-trained Paligemma model was implemented for bearing defect detection using AE signal images, demonstrating exceptional performance and accuracy. This approach highlights the critical importance of dataset quality, image resolution, and pre-training in LLM-based detection tasks. The findings establish a significant foundation for condition monitoring and fault diagnosis of rolling bearings in passenger ropeway systems. Corresponding vibration frequencies were evaluated numerically using the linear correlation coefficient and error value.

## 2. Materials and Methods

**Figure 1:**
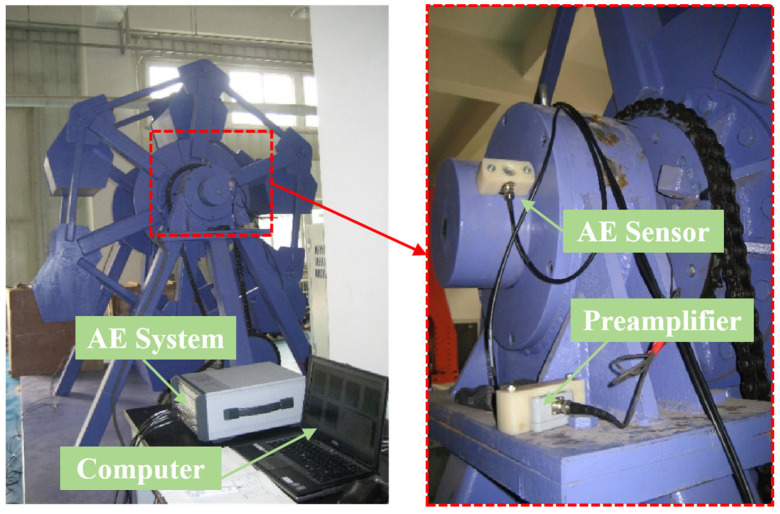
Laboratory rolling bearing testing device.

**Figure 2:**
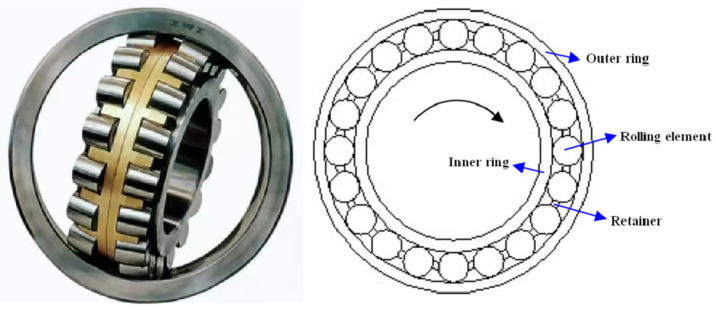
Testing rolling bearing (23138CA).

The experimental setup consisted of a rolling bearing test rig integrated with a digital multi-channel AE system (AMSY-6, Vallen-Systeme GmbH, Munich, Germany), including high-sensitivity sensors, preamplifiers, and a computer running specialized signal-processing software. The sensor was mounted on the shaft using a screw clamp with couplant lubrication, as shown in Figure 1. The test bearing was a 23138CA/W33 spherical roller bearing consisting of an inner ring, outer ring, double-row rolling elements, and cage assembly, as shown in Figure 2. The bearing had nominal dimensions of 190 mm bore diameter, 320 mm outside diameter, and 104 mm width, with a total weight of 34.4 kg. The component specifications were as follows: the inner ring had a thickness of 14 mm, the outer ring measured 20 mm in thickness, and each of the two rows contained 20 rolling elements with an end-face diameter of 30 mm and length of 40 mm.

**Figure 3:**
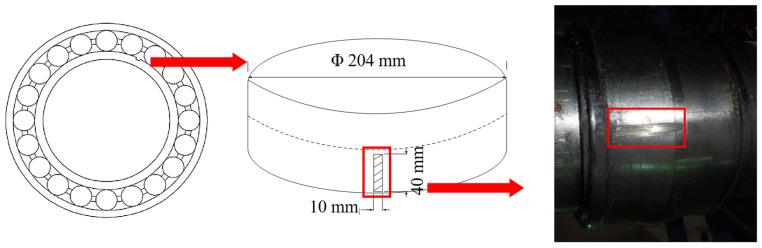
Location of the inner ring defect.

The AE data acquisition employed broadband VS45-H sensors (40–450 kHz bandwidth) and resonant VS150-RIC sensors (150 kHz peak frequency, 100 Hz–450 kHz range, Vallen Systeme GmbH, Wolfratshausen, Germany) to study signal spectrum characteristics, with 34 dB gain preamplifiers using 20–1000 kHz bandpass filters, a 10 MHz sampling rate, and 819.2 μs sample time. Based on field measurements indicating a background noise level of 39 dB, the threshold value was set to 45 dB to effectively eliminate laboratory noise interference in the AE monitoring system. A simulated rectangular wear defect (40 × 10 × 3 mm) was artificially introduced on the inner race, as shown in Figure 3. The ropeway was initiated under no-load conditions, with signal acquisition commencing upon reaching nominal operating speed. For both defective and intact bearings, AE signals were collected over 10 complete rotational cycles (at 0.32 rpm, 188 s/revolution), enabling comprehensive characterization of AE signatures in low-speed rolling bearings.

## 3. Results and Discussion

### 3.1. Result in the Laboratory

#### 3.1.1. Waveform and Spectrum Analysis of Rolling Bearings

**Figure 4:**
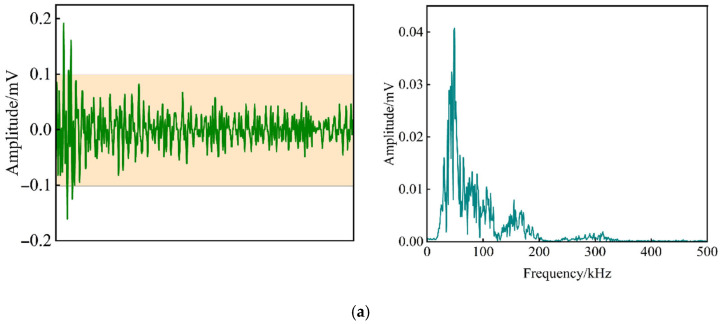

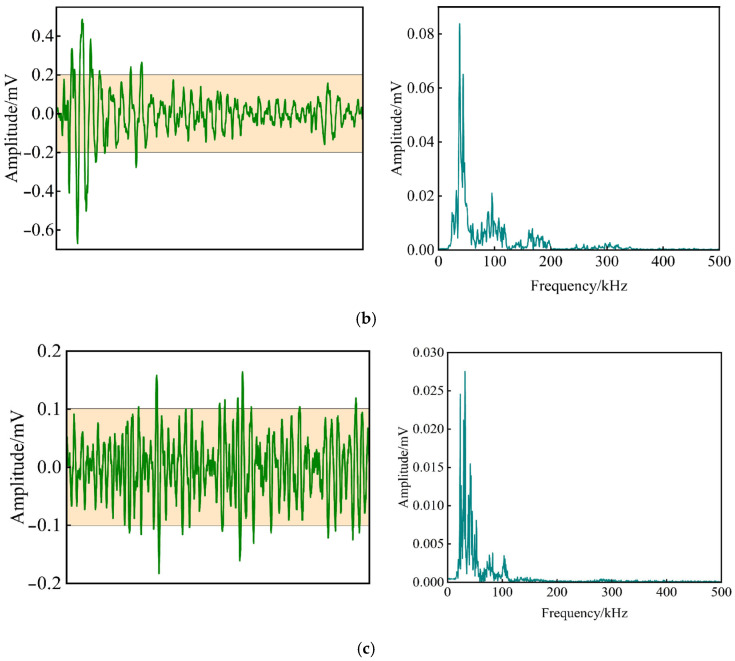
Waveform and spectrum analysis of sensor VS45-H: (**a**) AE signal of non-defective rolling bearing; (**b**) AE signal of defective rolling bearing; (**c**) AE signal of field testing.

**Figure 5:**
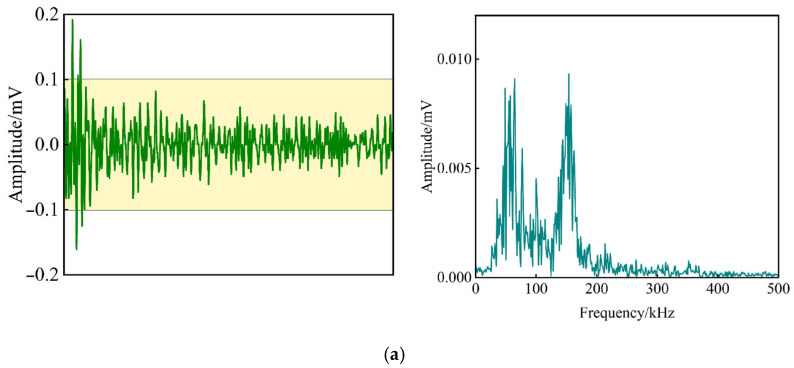

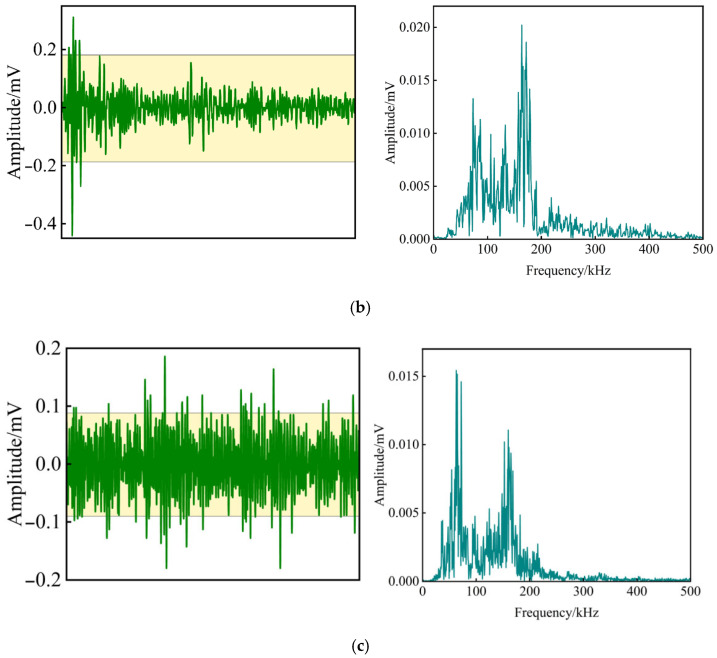
Waveform and spectrum analysis of sensor VS150-RIC: (**a**) AE signal of non-defective rolling bearing; (**b**) AE signal of defective rolling bearing; (**c**) AE signal of field testing.

This section conducts a comparative analysis of waveforms and spectra to examine the frequency characteristics of non-defective and defective rolling bearings, with AE signals collected from both broadband (VS45-H) and resonant (VS150-RIC) sensors. Results reveal that while the VS45-H sensors exhibit high sensitivity in the low-frequency range (20–190 kHz) with a dominant peak below 60 kHz, the VS150-RIC sensors effectively capture the non-defective bearing’s frequency distribution (40–180 kHz) with dual peaks at 60 kHz and 150 kHz, while also demonstrating heightened sensitivity to defective bearings through increased waveform amplitude and a notable spectral energy shift toward 150 kHz, ultimately proving the VS150-RIC sensors are more adept at detecting bearing defects, and thus, they are selected for subsequent analysis, see Figure 4 and Figure 5.

#### 3.1.2. Data Analysis for AE Characteristics of Rolling Bearings

The traditional parameter analysis method, widely adopted for its simplicity and practical applicability, effectively extracts acoustic emission characteristics from low-speed rotating rolling bearings through the statistical analysis of AE parameters, which is the approach utilized in this section to examine the operational behavior of the bearings.

##### Traditional Analysis of AE Parameters

Given the periodic rotational nature of rolling bearings, AE parameter features of single circle and multi-circle signals were analyzed, respectively.

(1) One-rotating-circle signals

**Figure 6:**
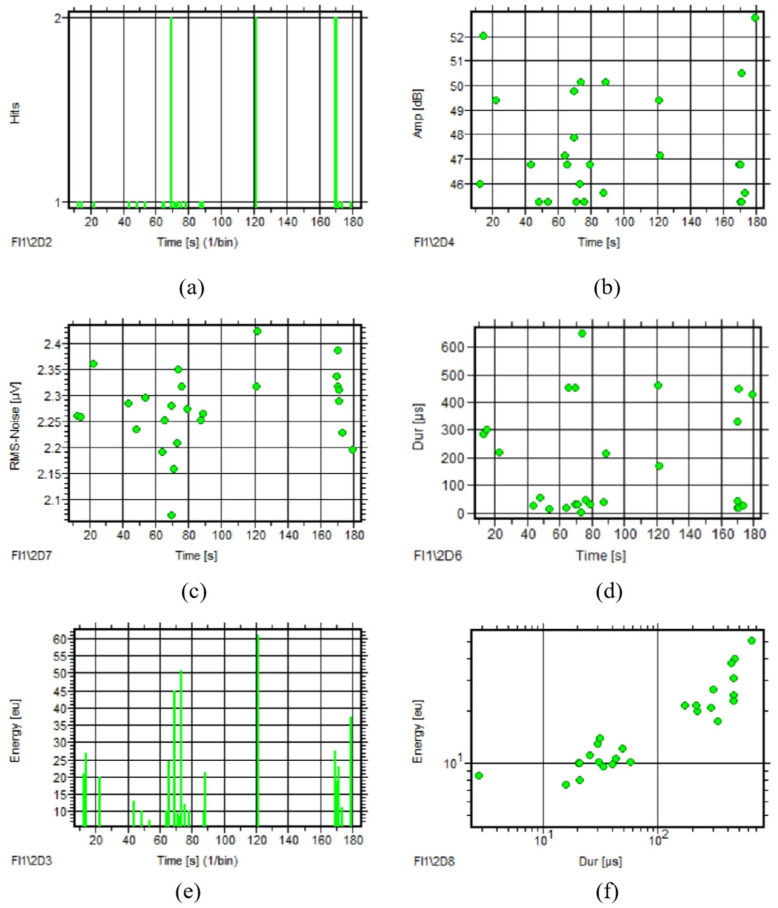
Parameter analysis of non-defective rolling bearing (1 rotating circle): (**a**) Hits–time; (**b**) Amp–time; (**c**) RMS–time; (**d**) Dur–time; (**e**) energy–time; (**f**) energy–Dur.

For one rotating circle of a non-defective rolling bearing, AE signals appeared occasionally with no obvious characteristics of the AE parameter courses. Also, there was one kind of signal through correlation analysis from the energy–duration (Dur) graph, as seen in Figure 6. The AE parameter distribution and concentration of the signals were obtained by distribution analysis, shown in Table 1.

**Figure 7:**
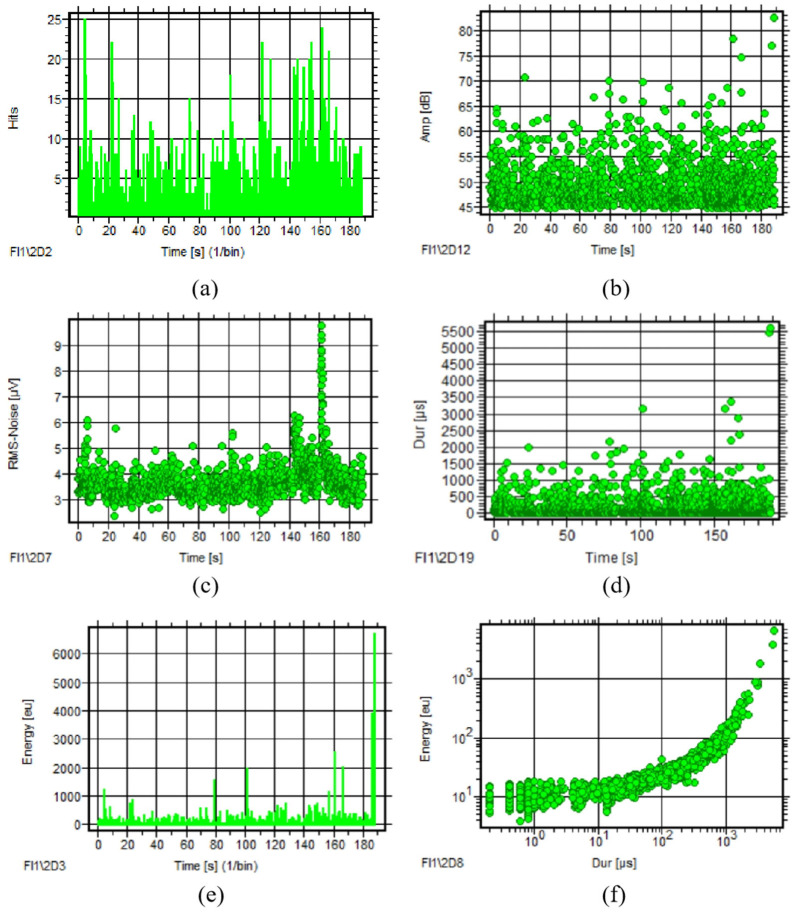
Parameter analysis of defective rolling bearing (1 rotating circle): (**a**) Hits–time; (**b**) Amp–time; (**c**) RMS–time; (**d**) Dur–time; (**e**) energy–time; (**f**) energy–Dur.

Figure 7 reveals a significant increase in hit counts and enhanced discernibility of other AE parameters in rolling bearings with inner race defects. There is only one obvious peak in the RMS course and energy course, but the Dur–time course has multiple irregular peaks. Figure 7f shows it has the same associated feature with non-defective rolling bearing, except the signals are increasing.

The AE parameter distribution of the defective rolling bearing is shown in Table 2. Compared with Table 1, the AE signals of the defective rolling bearing have higher amplitude, energy, and RMS. Only a few of them have larger counts and longer durations and rise time.

(2) Ten rotating circle signals

**Figure 8:**
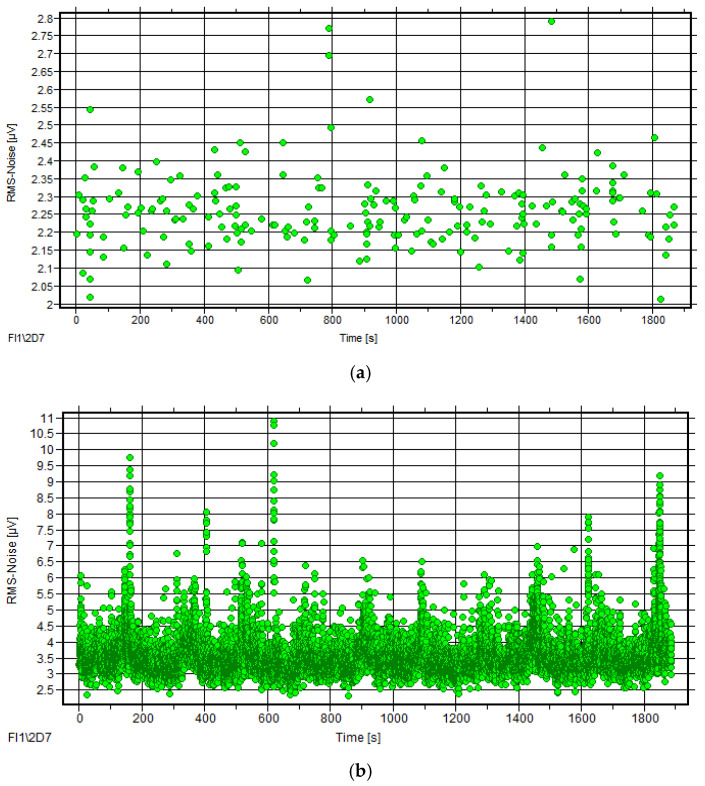

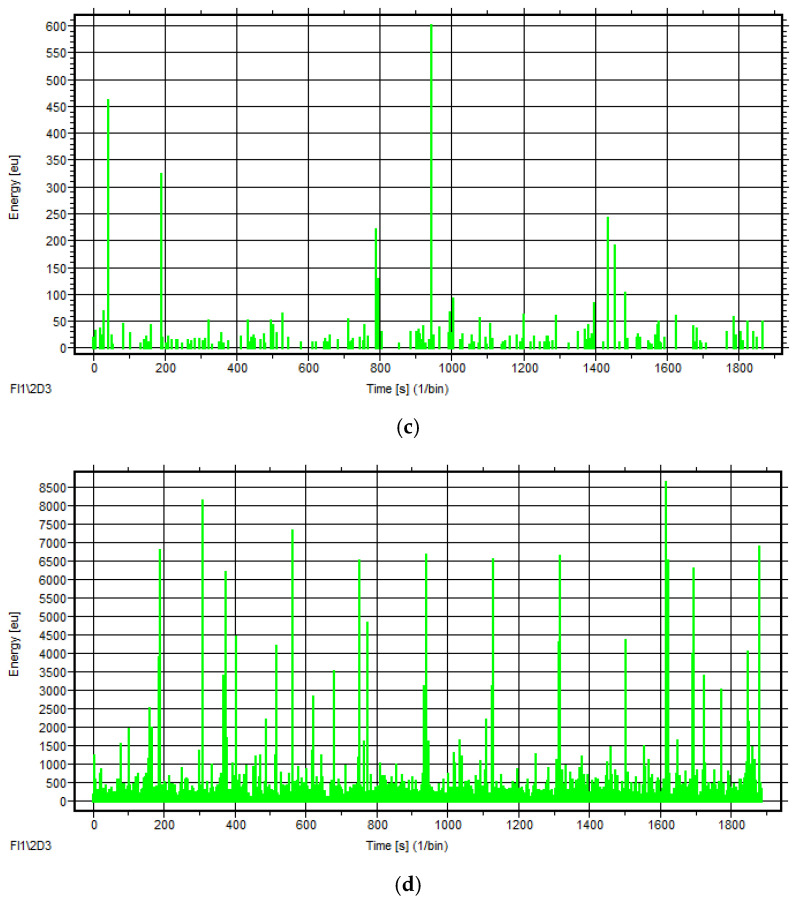
AE parameter courses of two bearings (10 rotating circles): (**a**) RMS–time of non-defective rolling bearing; (**b**) RMS–time of defective rolling bearing; (**c**) Energy–time of non-defective rolling bearing; (**d**) Energy–time of defective rolling bearing.

For one-rotating-circle signals, peaks appear in the AE parameter courses of the defective rolling bearing in Figure 7. In order to explain the appearance of peaks, a comparison between the non-defective rolling bearing and the defective rolling bearing was carried out by AE parameter courses of 10 rotating circles. A lot of peaks appear in all the AE parameter courses when the rolling bearing has a defect on the inner race. Only the peaks in the figures of RMS–time and energy–time present regularity, which is more obvious in the RMS–time, as seen in Figure 8. This periodicity in RMS values, which is more pronounced than in energy measurements, physically originates from transient stress waves generated during periodic roller-defect interactions and microfracture emissions at the defect edges when the bearing operates. The clearer periodicity observed in RMS compared with energy parameters occurs because the RMS values more effectively isolate these transient impact events from the continuous background noise and frictional components that dominate the energy measurements. These findings directly address the underlying physical processes connecting the defect to AE signal generation, as the 188 s peak interval provides clear evidence that the detected signals are synchronized with the bearing’s rotational kinematics and defect contact events.

The preceding AE parameter analysis demonstrates that root-mean-square (RMS) values exhibit both elevated amplitude and distinct periodicity in defective rolling bearings. These attributes establish RMS as the most reliable indicator for condition monitoring and fault diagnosis in passenger ropeway bearing systems.

##### Statistical Analysis of AE Parameters

The statistical analysis method can extract useful information quickly from a large quantity of signals. In this study, the AE parameters were calculated according to the rotating circle of the rolling bearing. For each circle, the sum of Hits and the mean value of other AE parameters were defined as the statistical parameters. For example, the mean value of RMS is the sum of RMS divided by the sum of Hits with signals of one rotating circle, abbreviated as RMS mean. Then, fitting curves of the statistical parameters with rotating circles were obtained by MATLAB R2023b.

**Figure 9:**
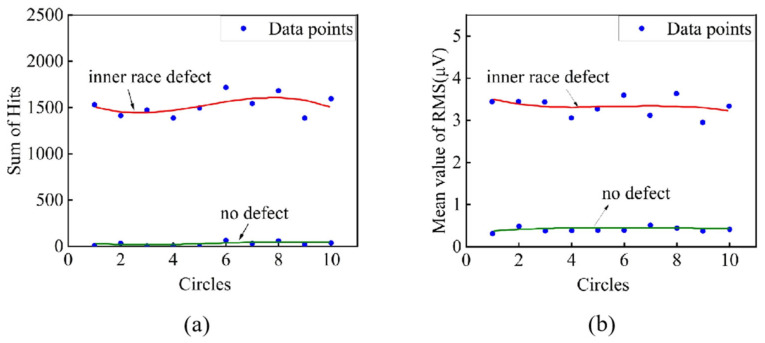
Fitting curves of the statistical parameters of two bearings: (**a**) the fitting curves of Hits sum; (**b**) the fitting curves of RMS mean.

Contrasting the statistical results of the non-defective bearing and the defective bearing, we found that the two bearings can be clearly differentiated in fitting curves of Hits sum and RMS mean, as seen in Figure 9. For these two statistical parameters, the fitting curves of the -defective bearing are significantly higher than the bearing with no defect, whereas other statistical parameters do not have this feature. Also, the RMS mean is more meaningful because it has eliminated the impact of the Hits sum increase. Therefore, the RMS mean can be used as the characteristic value for defect detection of rolling bearings.

### 3.2. Result in the Field

Building upon these findings, acoustic emission testing for condition monitoring of rolling bearings was conducted on dozens of passenger ropeways in China, with each ropeway’s rolling bearings tested at least biennially. The condition assessment was performed using the AE signal analysis methodology proposed in Section 2. Results indicated that most ropeway rolling bearings maintained satisfactory operational conditions, while a few exhibited significant elevations in AE characteristic parameters; consequently, two representative cases with divergent test outcomes are presented herein. Field testing was conducted using the digital multi-channel AMSY-6 AE system equipped with VS150-RIC sensors to monitor the passenger ropeway bearings under no-load conditions, with a preamplifier gain of 34 dB, a 20–1000 kHz bandpass filter, a sampling rate of 10 MHz, a sampling duration of 819.2 μs, and a threshold of 45 dB.

#### 3.2.1. Results of AE Testing on Ropeway I

**Figure 10:**
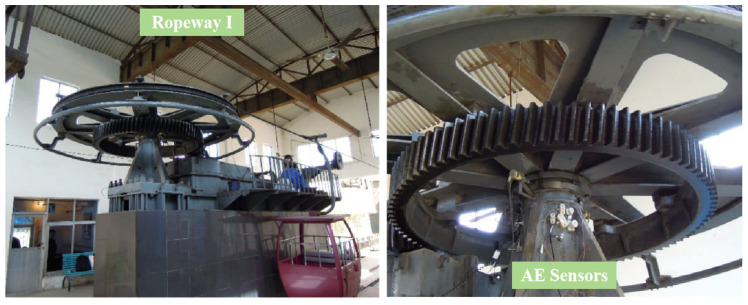
Ropeway I and sensor installation locations.

Ropeway I is located on a mountain with an elevation difference of 425 m and a horizontal distance of 843 m. The rolling bearings of the ropeway are self-aligning roller bearings of type GB/T 288 23152CC/W33, as seen in Figure 10. The rotating circle of the rolling bearings is 4.4 s, according to the line speed and wheel circumference of the ropeway.

The AE tests of the rolling bearings on the ropeway were carried out in 2022 and 2024. AE signals were acquired during the normal operation of the ropeway. AE parameters and statistical parameters were analyzed for the two tests.

##### AE Parameter Analysis

**Figure 11:**
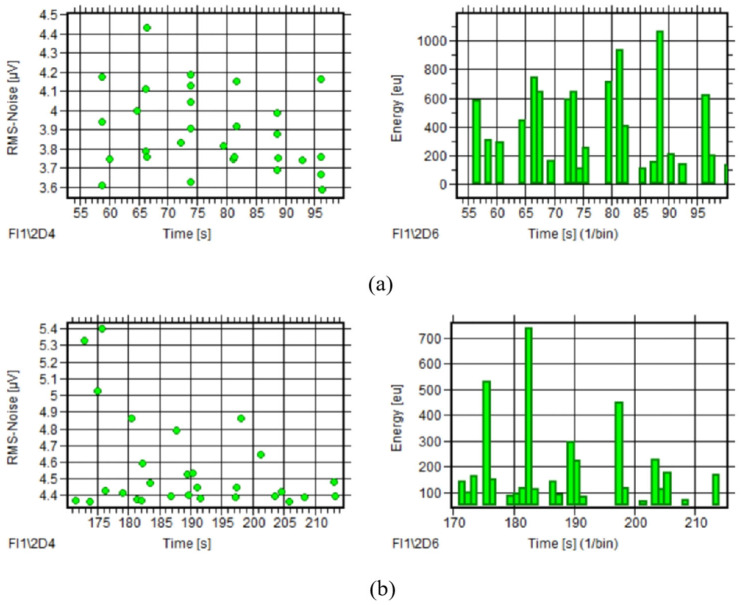
AE parameter courses of ropeway I (tests 2022 and 2024): (**a**) RMS–time and energy–time of test 2022; (**b**) RMS–time and energy–time of test 2024.

During normal ropeway operation, extensive AE signal data were collected, with comparative analysis of 10 rotation cycles from 2022 and 2024 tests revealing consistent temporal trends in RMS and energy parameters despite irregular signal distribution and an absence of periodic peak patterns, as seen in Figure 11, while all parameters maintained similar distribution ranges across both tests, as quantified in Table 3.

##### Statistical Analysis

**Figure 12:**
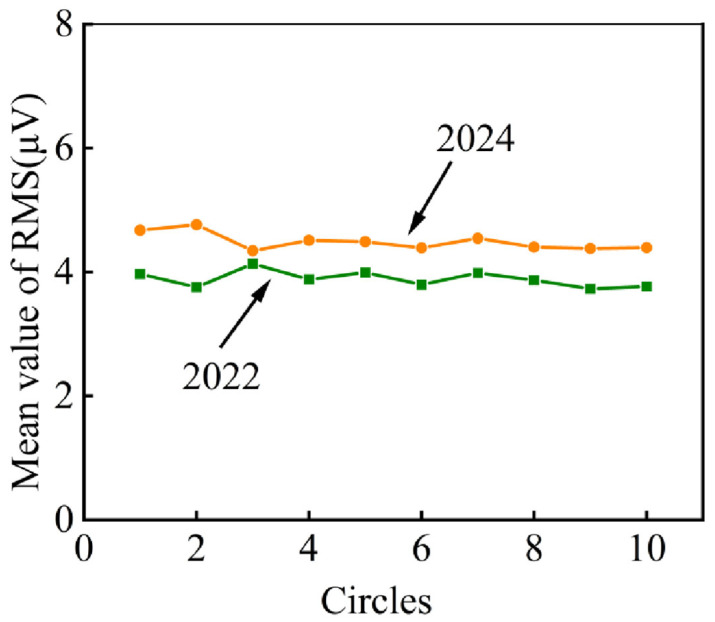
Fitting curves of RMS mean of ropeway I in 2022 and 2024.

Statistical analysis of ten rotation cycles revealed consistent RMS mean values between the two tests with no significant amplitude variations, as evidenced by the fitting curves of characteristic parameters versus rotation cycles shown in Figure 12.

Through comparative analysis using the methods above, it can be concluded that the AE signals acquired in 2022 and 2024 have the same AE characteristics. Consequently, there is no obvious change in health status of the rolling bearing on ropeway I.

#### 3.2.2. Results of AE Testing on Ropeway II

**Figure 13:**
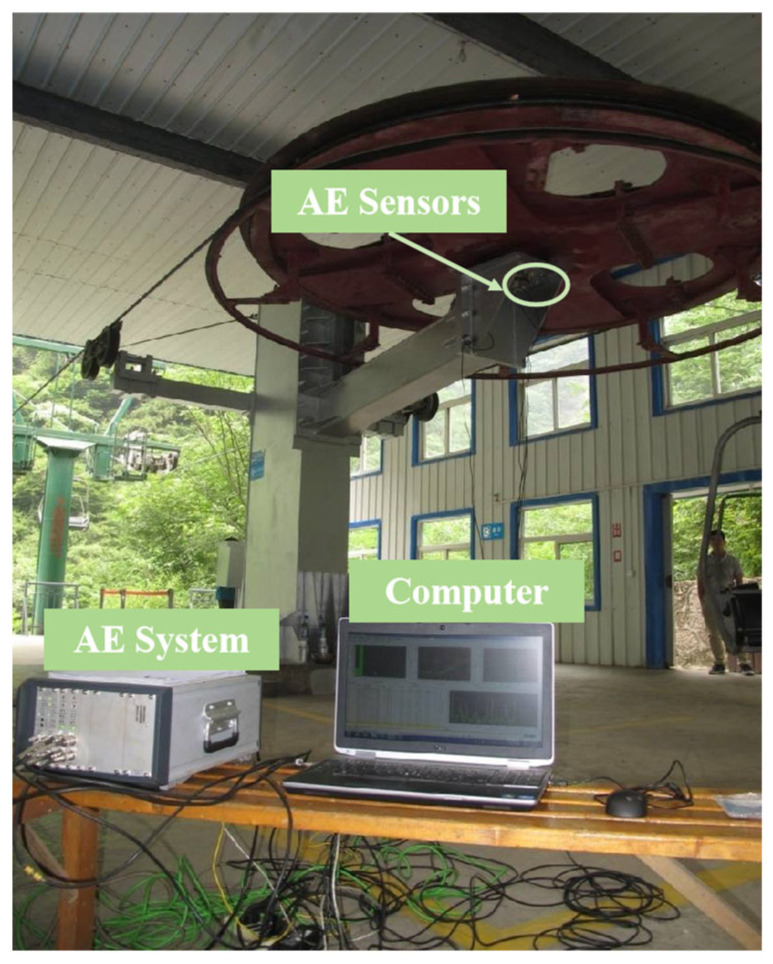
Ropeway II and sensor installation locations.

Ropeway II, located within a zoo, features an elevation difference of 3.4 m and a horizontal span of 385 m, utilizing rolling bearings of model GB297-64 7536E, which complete one rotation every 12.2 s based on the ropeway’s linear velocity and wheel circumference, as seen in Figure 13. The rolling bearings were subjected to acoustic emission testing in both 2022 and 2024, during which extensive AE signals were collected during normal operation and subsequently analyzed using identical methodologies for both AE parameters and statistical characteristics.

##### AE Parameter Analysis

**Figure 14:**
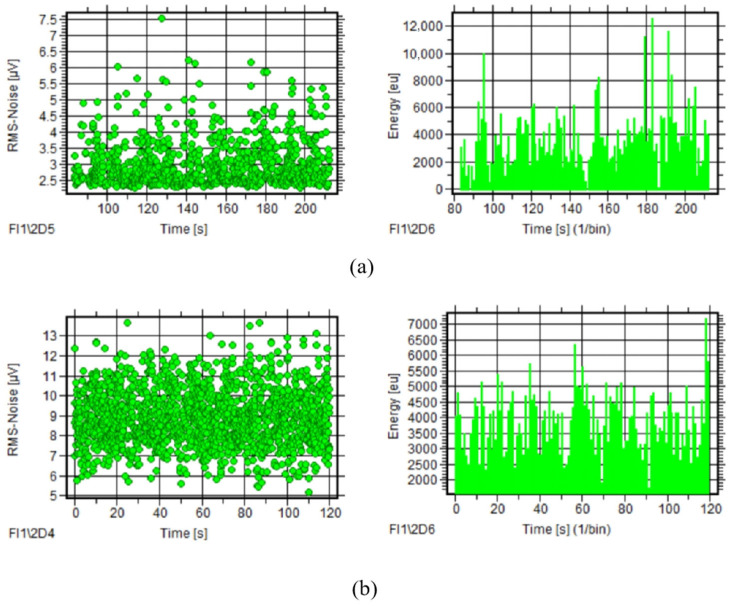
AE parameter courses of ropeway II (tests 2022 and 2024): (**a**) RMS–time and energy–time of test 2022; (**b**) RMS–time and energy–time of test 2024.

From the AE signals acquired in tests in 2022 and 2024, 10 rotating circle signals were selected separately. Observing the AE parameter courses of the two tests, RMS and energy also have the same variation tendency along with time. The RMS–time and energy–time courses show more obvious regularity of the signals obtained in 2024. The RMS has a clear feature of periodic peaks in which the peak interval is close to the bearing rotating circle, as seen in Figure 14. Table 4 gives the parameter distribution of AE signals of the two tests. Compared with the signals of 2024, only the values of RMS increase significantly, whereas the other parameters’ range is similar to the signals of 2022.

##### Statistical Analysis

**Figure 15:**
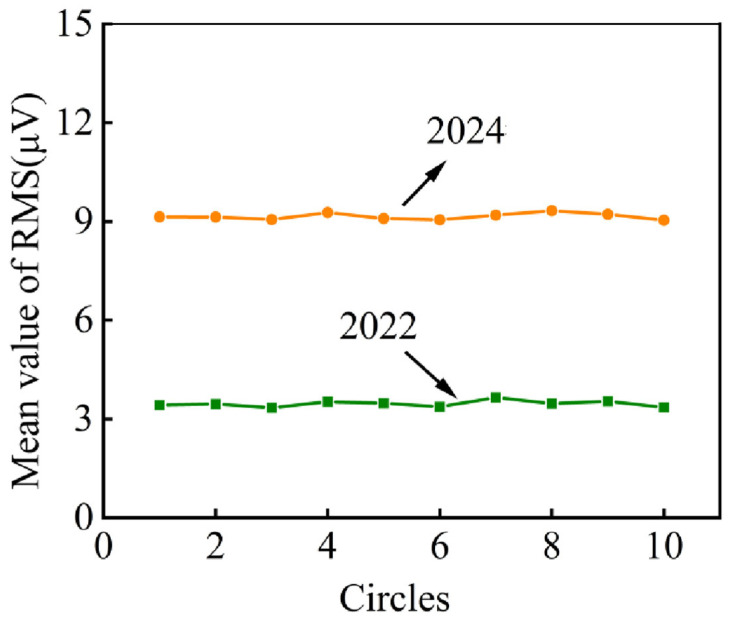
Fitting curves of RMS mean of ropeway II in 2022 and 2024.

Figure 15 shows the fitting curves of the RMS mean with rotating circles of the AE signals obtained in 2022 and 2024. The values of the RMS mean fluctuate within a certain scope with no obvious change in each test. Contrasting the two tests, the values of the RMS mean in 2024 are about twice as many as in 2022, which means there is a serious increase in the characteristic value.

The comparative analysis of both tests revealed that the RMS characteristic parameter exhibited periodic peak features and value escalation, with its mean value progressively increasing after two years of ropeway operation, demonstrating AE characteristics consistent with laboratory-analyzed defective rolling bearings and suggesting that ropeway II’s bearings show early damage indicators warranting immediate inspection.

### 3.3. LLM-Based Bearing Wear Detection

Based on the above experimental investigation regarding bearing wear defect, this study proposes an LLM-based bearing wear detection approach. The method uses the time-domain monitoring curve image of the AE signal as input and generates the wear feature area on the calibrated time-domain monitoring curve image, thereby enabling automated identification of the presence of wear in the bearing. The LLM model utilized in this study is the Paligemma model, which is publicly available from Google. This model is capable of accurately understanding and interpreting image content. Using this large model and utilizing the AE signal data of bearings with and without wear, the wear and non-wear features were manually annotated to establish a benchmark dataset for developing the LLM-based bearing wear detection model.

For the experimental dataset, a total of 350 AE signal images were collected, comprising 200 images of non-defective rolling bearings and 150 images of defective rolling bearings. The open-source tool YoloLabel was employed to annotate these AE signal images, with the label “non defect” assigned to non-defective bearings and “bearing defect” to defective ones. The annotated images and their corresponding labels were then organized into a benchmark development dataset to support the training and evaluation of the proposed LLM-based bearing wear detection model. To facilitate model development, the dataset was divided into two subsets: a training set and a testing set. The training set consisted of 280 images and was used during the learning phase of the model, while the testing set included 70 images and was utilized to evaluate the final performance of the trained model.

#### 3.3.1. Effect of the Image Size on the LLM Model

**Figure 16:**
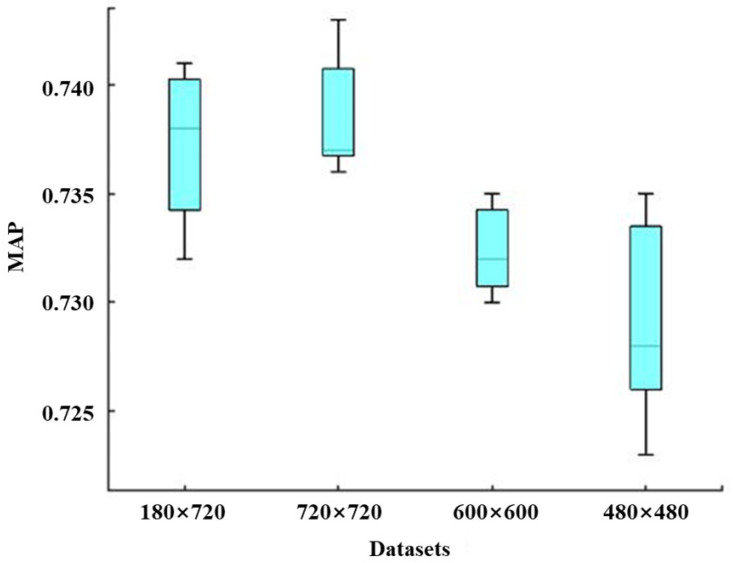
Boxplot of MAP concerning the Paligemma model developed by different image size datasets.

Figure 16 displays corresponding boxplots concerning Paligemma models developed by different developing datasets with image size (1280 × 720), (720 × 720), (600 × 600), and (480 × 480). From this figure, one can find that the whole MAP trend of the developed models decreases with the decline in developing image size. Specifically, although the max and min MAP values of models developed by image size 720 × 720 are all greater than those of models developed by 1280 × 720, the MAP median of models developed by 720 × 720 is greater than that of the models developed by 1280 × 720. Furthermore, the MAP values under 600 × 600 and 480 × 480 all have a certain decline compared with those under 1280 × 720 and 720 × 720, and the MAP under dataset 480 × 480 has also a certain decline relative to that under dataset 600 × 600. Overall, the drop in the image size of the developing dataset results in the MAP of the corresponding models dropping.

#### 3.3.2. Effect of the Noise Intensity on the LLM Model

**Figure 17:**
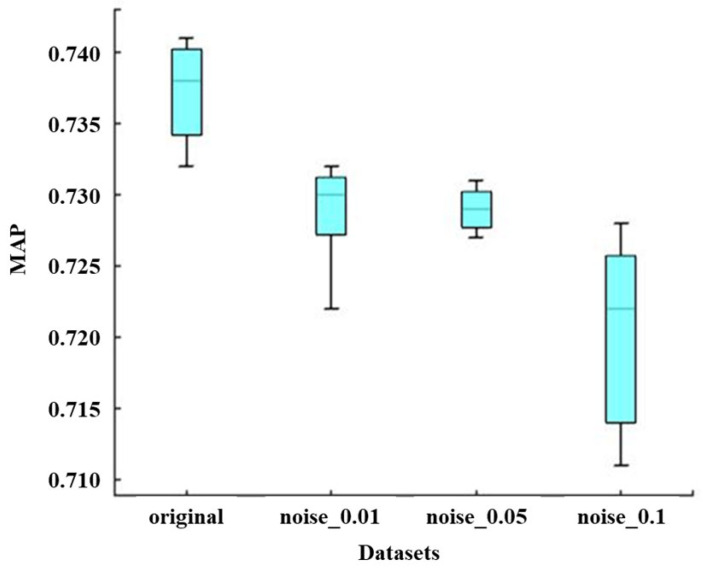
Boxplot of MAP concerning Paligemma models by noise intensities of different developed images.

Figure 17 illustrates boxplots of Paligemma models developed using datasets with image sizes of 1280 × 720, 720 × 720, 600 × 600, and 480 × 480. It can be observed from the figure that the overall MAP trend decreases as the image size of the training dataset decreases. Specifically, although the maximum and minimum MAP values of models developed using 720 × 720 images are both greater than those of models developed using 1280 × 720 images, the MAP median of models developed using 720 × 720 images is higher than that of models developed using 1280 × 720 images. Furthermore, the MAP values for 600 × 600 and 480 × 480 images exhibit a noticeable decline compared with those for 1280 × 720 and 720 × 720 images, with the MAP for 480 × 480 images being lower than that for 600 × 600 images. Overall, a decrease in the image size of the training dataset leads to a reduction in the MAP of the corresponding models.

#### 3.3.3. Validation and Comparison of LLM-Based Bearing Wear Detection

**Figure 18:**
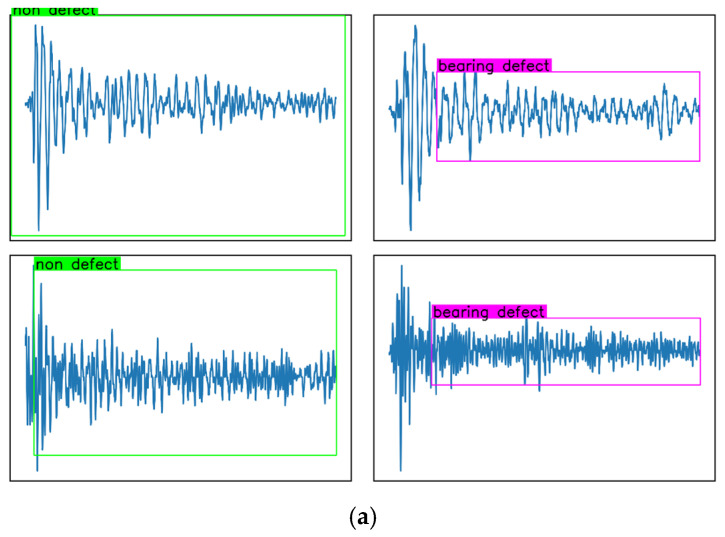

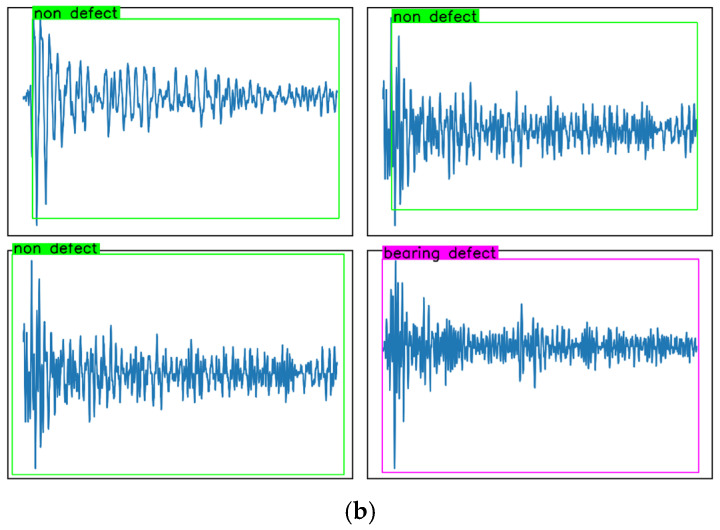
Comparison of detection results generated by the pre-trained Paligemma model and the non-pre-trained Paligemma model: (**a**) pre-trained Paligemma model; (**b**) non-pre-trained Paligemma model.

A comparison of the detection results generated by the pre-trained and non-pre-trained Paligemma models, as seen in Figure 18, reveals a clear performance advantage of the pre-trained model. The pre-trained Paligemma model (a) demonstrates superior capability in accurately identifying both wear and non-wear features, which is evidenced by the clearer and more reliable localization of the annotated regions. Specifically, the pre-trained model not only detects wear features but also correctly recognizes non-wear features, thereby providing a balanced and robust detection performance. In contrast, the non-pre-trained Paligemma model (b) exhibits poor detection performance. Although it provides wear labels, these labels are generally inaccurate, and the model completely fails to identify non-wear features. These findings confirm that the pre-trained Paligemma model is better suited for bearing wear detection, as it leverages prior knowledge to achieve improved generalization and higher recognition accuracy.

## 4. Conclusions

This study investigated the AE characteristics of rolling bearings in passenger ropeway systems through comprehensive laboratory experiments and field testing, providing valuable insights for condition monitoring and fault diagnosis. The key findings are summarized as follows.

(1) AE signals from rolling bearings were systematically compared using two distinct sensors. Broadband sensor VS45-H captured dominant AE signal components below 60 kHz, whereas resonant sensor VS150-RIC detected frequency distributions between 40 kHz and 180 kHz, with peaks at 60 kHz and 150 kHz. Defective bearings exhibited a pronounced energy increase near 150 kHz, validating the effectiveness of resonant sensors for defect detection.

(2) Non-defective rolling bearings produced sporadic AE signals with no distinct patterns, while bearings with inner race defects exhibited significantly higher amplitude, energy, and RMS values. The defective bearings also generated a markedly increased number of signals, highlighting the sensitivity of the AE technology to early-stage damage. The RMS–time course of the defective bearings displayed periodic peaks aligned with the bearings’ rotational cycles, demonstrating its reliability for fault detection. Statistical analysis further confirmed that the mean RMS value serves as a robust characteristic parameter for identifying bearing defects, eliminating the influence of signal quantity variations.

(3) Field tests on multiple passenger ropeways demonstrated the practical applicability of AE monitoring. Ropeway I showed stable AE characteristics over two years, indicating healthy bearing conditions, while ropeway II revealed escalating RMS values and periodic peaks. The results indicate that the bearing in ropeway II is in an early-stage damage condition, warranting reduced inspection intervals and timely adjustment of maintenance strategies.

(4) An LLM-based approach for bearing wear detection by using time-domain monitoring curve images of AE signals is proposed. The results demonstrate that both the image size and noise intensity of the training dataset significantly influence the detection accuracy of the model. Specifically, larger image sizes result in improved Mean Average Precision (MAP), while higher noise intensities reduce detection accuracy. Furthermore, the pre-trained Paligemma model achieved substantially higher performance than its non-pre-trained counterpart, accurately detecting both wear and non-wear features. These findings underscore the importance of dataset quality and the effectiveness of pre-training in improving the generalization and accuracy of LLM-based models for bearing wear detection.

## Figures and Tables

**Table 1:** Parameter distribution of AE signals of the non-defective rolling bearing.

AE Parameter	Distribution	Concentration	AE Parameter	Distribution	Concentration
Amp (dB)	45~53	45~51	Dur (μs)	1~650	1~110
Energy (eu)	1~60	1~35	Rise time (μs)	1~150	1~50
Counts	1~95	1~10	RMS (μV)	2~3	2~2.5

**Table 2:** Parameter distribution of AE signals of defective rolling bearing.

AE Parameter	Distribution	Concentration	AE Parameter	Distribution	Concentration
Amp (dB)	45~90	45~60	Dur (μs)	1~5500	1~1000
Energy (eu)	1~6000	1~500	Rise time (μs)	1~350	1~50
Counts	1~25	1~10	RMS (μV)	2~11	2~6

**Table 3:** Parameter distribution of AE signals of ropeway I (tests 2022 and 2024).

AE Parameter	2022	2024	AE Parameter	2022	2024
Amp (dB)	45~66	45~62	Dur (μs)	1~6000	1~5000
Energy (eu)	1~700	1~650	Rise time (μs)	1~3000	1~2500
Counts	1~350	1~400	RMS (μV)	3~4.5	4~5

**Table 4:** Parameter distribution of AE signals of ropeway II (tests 2022 and 2024).

AE Parameter	2022	2024	AE Parameter	2022	2024
Amp (dB)	45~62	45~68	Dur (μs)	1~7000	1~5000
Energy (eu)	1~8000	1~6000	Rise time (μs)	1~2000	1~1500
Counts	1~300	1~250	RMS (μV)	2~6	6~12

## Data Availability

All data are available from the authors upon reasonable request.
